# The Lightweight Anchor Dynamic Assignment Algorithm for Object Detection

**DOI:** 10.3390/s23146306

**Published:** 2023-07-11

**Authors:** Ping Han, Xujun Zhuang, Huahong Zuo, Ping Lou, Xiao Chen

**Affiliations:** 1 School of Information Engineering, Wuhan University of Technology, Wuhan 430070, China; 2 Wuhan Chuyan Information Technology Co., Ltd., Wuhan 430030, China

**Keywords:** object detection, positive and negative samples, anchor assignment, aspect ratio, loss aware, self adaptive, loss of anchor

## Abstract

Smart security based on object detection is one of the important applications of edge computing in IoT. Anchors in object detection refer to points on the feature map, which can be used to generate anchor boxes and serve as training samples. Current object detection models do not consider the aspect ratio of the ground-truth boxes in anchor assignment and are not well-adapted to objects with very different shapes. Therefore, this paper proposes the Lightweight Anchor Dynamic Assignment algorithm (LADA) for object detection. LADA does not change the structure of the original detection model; first, it selects an equal proportional center region based on the aspect ratio of the ground-truth box, then calculates the combined loss of anchors, and finally divides the positive and negative samples more efficiently by dynamic loss threshold without additional models. The algorithm solves the problems of poor adaptability and difficulty in the selection of the best positive samples based on IoU assignment, and the sample assignment for eccentric objects and objects with different aspect ratios was more reasonable. Compared with existing sample assignment algorithms, the LADA algorithm outperforms the MS COCO dataset by 1.66% over the AP of the baseline FCOS, and 0.76% and 0.24% over the AP of the ATSS algorithm and the PAA algorithm, respectively, with the same model structure, which demonstrates the effectiveness of the LADA algorithm.

## 1. Introduction

Due to the advantages of edge computing in terms of latency, bandwidth, and security, it provides more effective technical support for many applications that require real-time security. A security system is one of the applications for edge computing in IoT, where the core end device is a camera for surveillance and smart security. Intelligent security systems require accurate and real-time processing of images and video from cameras, which poses a challenge to existing object detection technologies.

The training samples in object detection use anchors, which are feature points on the feature map that can be used for classification and regression. In anchor-based detection models (e.g., RetinaNet [1]), multiple anchor boxes can be tiled with anchors as the center, while in anchor-free models (e.g., FCOS [2]), anchors can be used as anchor points to directly predict regression boxes. In recent years, although great breakthroughs have been made in object detection, there are still some shortcomings in the definition of positive and negative samples in current object detection. As the aspect ratio of the ground-truth (GT) boxes are not fixed, it is difficult for the ordinary center prior to select suitable anchors as positive samples. Moreover, the distribution of objects within a GT box is uncertain, there is often a large amount of background within the labeled GT box, and there may also be interference, such as occlusion. In this case, the traditional approach based on fixed Intersection-over-Union (IoU) assignment [1] does not select better positive samples, which poses a challenge for positive and negative sample assignment. To address the problems posed by fixed IoU, [3] uses the statistical characteristics of IoU between the candidate anchor boxes and a GT box to give the algorithm a different IoU threshold. Ref. [4] proposes a new anchor quality evaluation scheme and uses the quality scores of anchors fitting a probability distribution to achieve a truly dynamic assignment; however, the computational effort is large and reduces the training speed. In summary, the shortcomings of existing positive and negative sample assignment algorithms include:

1. The aspect ratio of the GT boxes is not considered. For objects with a large difference in length and width, a square area is still used as the center region when selecting candidate samples.
2. The actual content of the anchor box intersecting the GT box is not considered. If an anchor box has a large IoU with the GT box, it does not mean that this anchor box can get a prediction box that also has a large IoU with the GT box.
3. To determine positive and negative samples, complex models are used, which leads to long training times and heavy hardware costs for accuracy gains, increases the difficulty of training, and fails to adapt to the demands of edge computing.

The positive and negative sample assignment algorithm is a key factor affecting the object detection model. Therefore, finding a better sample assignment algorithm has become a popular research topic. This paper proposes a new positive and negative sample assignment strategy, called Lightweight Anchor Dynamic Assignment (LADA), to address the above-mentioned problems in existing assignment algorithms. This algorithm has three main advantages over existing assignment algorithms:

1. The usage of a new center prior called the Equally Proportional Center Prior (EPCP), which takes full advantage of the aspect ratio of GT boxes to select anchors that are more representative of the object’s characteristics.
2. The usage of a dynamically varying Combined Loss of Anchor (CLA), which is more representative of the anchors’ quality than the traditional IoU and takes full account of the actual distribution of objects within the GT box.
3. The proposition of a Dynamic Loss Threshold (DLT) to avoid high computational costs and to dynamically filter out anchors with smaller loss as positive samples in a more efficient way.

## 2. Related Work

### 2.1. Anchor-Based and Anchor-Free Detectors

Object detection is a fundamental yet challenging task in computer vision, requiring the model to predict a bounding box with a category label for each target object in the image. Currently, deep-learning-based object detection algorithms dominate and can be divided into anchor-based and anchor-free approaches according to whether an anchor box is predetermined. Anchor-based object detection algorithms can be classified into two-stage methods (e.g., R-CNN [5], Fast R-CNN [6], Faster R-CNN [7], and R-FCN [8]) and single-stage methods (e.g., SSD [9] and RetinaNet [1]) according to whether candidate boxes are generated or not. These methods first tile a large number of pre-defined anchor boxes on the image, then predict the category, refine the coordinates of these anchor boxes one or more times, and finally output these refined anchor boxes as prediction boxes. The two-stage methods are slow but accurate; however, the advent of FPN [10] and Focal Loss [1] has enabled single-stage methods to achieve the same accuracy as two-stage methods at a faster speed.

Anchor-based object detection methods require artificially setting parameters such as the scale and aspect ratio of the anchor box according to the characteristics of the dataset, resulting in parameter sensitivity and poor generalization of the detector. Therefore, anchor-free detectors have gradually become a research hotspot. Anchor-free detectors can be divided into center-based methods (e.g., FCOS [2]) and keypoint-based methods (e.g., CornerNet [11] and ExtremeNet [12]). Anchor-free detectors can directly predict objects without pre-defined anchor boxes, eliminating the hyperparameters associated with anchor boxes and achieving similar performance to anchor-based detectors, while also giving them more potential in terms of generalization capability.

The anchor point In FCOS is equivalent to the center of the anchor box in RetinaNet. An anchor point and the corresponding anchor box both correspond to the same location on the feature map. Therefore, they are collectively referred to as an anchor in this paper when there is no need to distinguish between an anchor point and anchor box.

### 2.2. Loss Function in Object Detection

Losses in object detection models typically include classification losses and regression losses, corresponding to the classification and regression branches, respectively. Calculating the loss based on the ground truth and the predicted values output by the detection head allows back-propagation to update the model parameters. The main classification losses commonly used in object detection models are OHEM [13], Focal Loss [1], GHM [14], Quality Focal Loss [15], and Varifocal Loss [16], and the main regression losses are IoU Loss [17], Distribution Focal Loss [15], GIOU Loss, and DIoU and CIoU Loss [18]. In addition, some object detection models add a parallel auxiliary branch to the regression branch, such as the centerness branch in [2] and the IoU branch in [4,19,20]. These branches predict a centerness or IoU for each anchor and can also calculate the loss from the true value, which is usually calculated using binary cross entropy (BCE) loss.

### 2.3. Anchor Assignment in Object Detection

**Figure 1:**
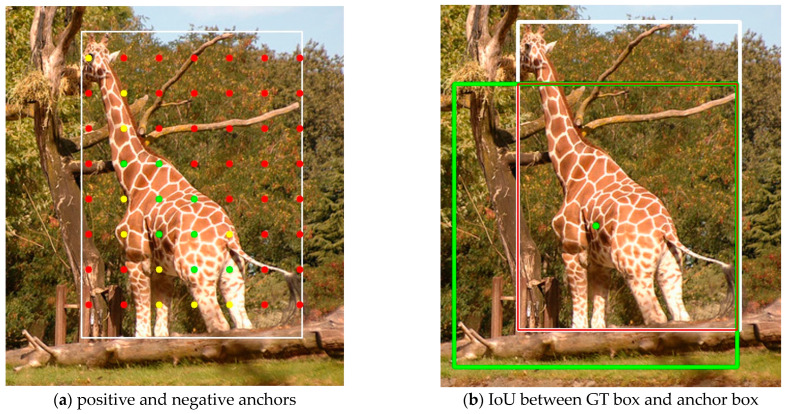
Example of anchors and the IoU of a GT box. (**a**) The white box is the GT box. The red dots are anchors that need to be assigned as negative anchors. The yellow dots are anchors that are better than red anchors, but not optimal positive anchors. The green dots are anchors which are expected to be assigned as positive anchors. (**b**) The white box is the GT box, and the green box is one of the anchor boxes of the green anchor. The red box is the intersection area between these two boxes.

Positive and negative sample assignment refers to the process of determining whether each anchor should be assigned as a positive or negative sample during training, a process also known as training sample selection, anchor assignment, and label assignment. Anchor assignment during training is an important factor affecting the performance of the object detection model. Figure 1a shows the anchor inside the GT box. The white box is the GT box and the different colors of anchors represent the high or low quality of the anchors. The red anchors are located in the background rather than the object and should be classified as negative anchors. The yellow anchors contain part of the object information and hence are not optimal positive anchors. Green anchors contain rich object information and should be used as positive anchors. Anchor assignment is used to pick out the green anchors and avoid the red anchors. The white box in Figure 1b is the GT, the green box is one of the anchor boxes corresponding to the green anchor, and the red box is their intersection area. By calculating the proportion of the intersection area to the concatenation of the two boxes, the IoU of the anchor box and the GT box can be obtained. We hope that the IoU of the predicted box and the GT box is as large as possible, rather than the IoU of the anchor box and the GT box.

Traditional positive and negative sample assignment strategies use manually designed hard assignments. Anchor-based detection models (e.g., RetinaNet [1]) empirically lay nine anchor boxes of different scales and aspect ratios at each location, and use a fixed IoU threshold to classify the anchor boxes into positive, ignored, and negative samples. This assignment requires multiple anchor boxes to be tiled per location, with many hyperparameters and a large computational cost. Anchor-free detection models (e.g., FCOS [2]) discard the tiling of anchor boxes and assign positive and negative samples using spatial constraints (i.e., restricting positive anchor points to inside the GT boxes) and scale constraints (i.e., setting a fixed maximum regression range for each feature layer). The subsequent FCOS [21] uses center sampling on top of the original constraints. The shortcoming that the above hard-assignment approaches all have in common is that they do not take into account the fact that the most meaningful anchors are not equally distributed within the GT boxes for objects of different sizes, shapes, or occlusion conditions, and the conditions for dividing positive and negative samples are not the same.

Due to the inadequacy of hard-assignment approaches, many algorithms for adaptively assigning anchors have emerged. For example, MetaAnchor [22] and Guided Anchoring [23] argue that tiled anchor boxes are not optimal. MetaAnchor uses the anchor function to dynamically generate anchor boxes from arbitrary customized prior boxes, thus changing the shape of anchor boxes during training. Guided Anchoring proposes the Guided Anchoring Region Proposal Network (GA-RPN), which generates anchor boxes through additional network modules to better fit the distribution of various objects. Both MetaAnchor and Guided Anchoring modify the structure of the model itself to varying degrees. FreeAnchor [24] defines the training process as one of maximum likelihood estimation based on classification loss and regression loss, and constructs a candidate set of anchors for each GT box. However, when a GT box has many high-quality anchors, this approach does not match the GT box well to the appropriate anchor.

ATSS [3] uses the sum of the mean and standard deviation of IoU values from a set of the closest anchors as the dynamic threshold. However, as the anchor boxes are constant, for the same GT box, this threshold is invariant during the training process. Moreover, using L2 distance is not reasonable for some GT boxes with a large difference in length and width. NoisyAnchors [25] proposes cleanliness scores, using soft labels and re-weighting based on classification and localization losses, which mitigates the effect of noise in the anchors but fixes the number of positive anchors throughout the training process. MAL [26] uses linear scheduling to reduce the number of positive samples as training proceeds but is prone to fall into suboptimal solutions and requires heuristic feature perturbations to mitigate this issue. PAA [4] assumes that the joint loss distribution of positive and negative samples follows the Gaussian distribution, and uses the Gaussian Mixture Model (GMM) to cluster the candidate positive samples to obtain the final positive samples. This approach does not make use of the shape of the GT box. In addition, the GMM requires continuous iteration, which is computationally intensive and extends the training time.

## 3. Methods

### 3.1. Equally Proportional Center Prior

The center region containing anchors is the area that is in the center of the GT box. The general sample assignment algorithms use the anchors of the center region as positive samples directly or as candidate positive samples for the next filtering step. In this paper, this step is referred to as center prior (CP). FCOS [2] directly selects anchors in the center region of a square as positive samples; ATSS [3] selects candidate positive samples based on L2 distance, so its center region is close to circular. However, instead of directly using anchors within the center region as positive samples, ATSS processes them further, and considers anchors outside the GT box to be poorer anchors. Both FCOS and ATSS, as well as the EPCP proposed in this paper, ensure that the positive anchors are inside the GT box.

In theory, all anchors within the GT box have the potential to be positive samples. However, in most cases, especially in the early stages of training, the anchors in the center of the object are more conducive to the training of the model, so the choice of the center region should be as reasonable as possible. FCOS directly selects the anchors in the small center region as positive samples, which leads to the model focusing too much on the central anchors; ATSS selects k candidate samples at L2 distance in each feature layer, which also only selects samples that are more clustered in the center region of the GT box. For some objects that are not completely in the center, it is difficult for these two methods to assign better anchors. However, if the center region is simply expanded, many anchors containing a lot of noise will be introduced, which will affect the performance of the detection model to some extent. In addition, FCOS and ATSS select candidate samples without considering the GT box’s shape at all. For some GT boxes with a large difference in length and width, the selection of anchors by FCOS or ATSS is not suitable.

To solve this problem, this paper proposes the EPCP. Different from the traditional center prior, EPCP determines the center region according to the aspect ratio of each GT box. For objects with similar length and width, the center region using EPCP is close to square, which is not much different from the center region using the traditional center prior. However, for objects with a large difference in length and width, the center region is rectangular and contains most of the anchor points that can represent the features of the object because the aspect ratio of the center region is the same as that of the GT box.

Specifically, the process of using EPCP to determine the center region is as follows: assume that all GT boxes in an image are the set G, and g is one of the GT boxes, i.e., g∈G. The length and width of g are H and W, respectively, the center coordinates are (x,y), and the stride from the feature layer to the original image is S, so the two distances from the center of the GT box to the left boundary and the upper boundary of the central area can be determined as shown in Equations (1) and (2), respectively:

(1) $$X_{s}=\frac{r\times S\times W}{R},$$

(2) $$Y_{s}=\frac{r\times S\times H}{R},$$

**Figure 2:**
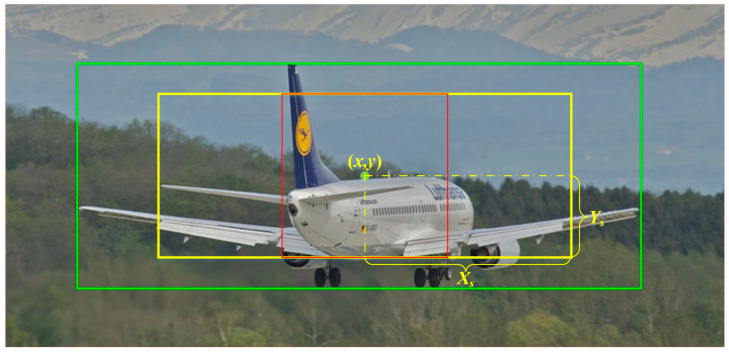
Center areas of EPCP (yellow box) and CP (red box) in a GT box (green box).

In these equations, r is a hyperparameter, and R=min(H,W). From these two distances, the coordinates of the four vertices of the center region with EPCP can be determined as $\left(x-X_{s},y-Y_{s}\right)$, $\left(x+X_{s},y-Y_{s}\right)$, $\left(x-X_{s},y+Y_{s}\right)$, and $\left(x+X_{s},y+Y_{s}\right)$, respectively. Through EPCP, the short side of the center region of each GT box is 2×r×S, and the center region maintains the same aspect ratio as the GT box. Figure 2 shows the center region obtained from EPCP; in this figure, the green box is the GT box, the yellow box is the center region of EPCP with the same aspect ratio as the GT box, and the red box is the center region of CP. The red box is still square when the GT differs greatly in length and width.

To ensure that the center region can cover all anchors suitable as positive samples as much as possible, this paper sets the hyperparameter r of the center region to 2.5, which is only 1.5 in the center sampling of FCOS. The area of the center region of FCOS is ${\left(2\times r\times S\right)}^{2}=9\times S^{2}$, while the shortest side of the central area of EPCP is 2×r×S=5S, so its minimum area is $25\times S^{2}$. By increasing the area of the center region, most of the anchors that may become positive samples are in the center region. In addition, better positive anchors will not be missed for some objects with a large difference in length and width, such as buses, giraffes, tennis rackets, toothbrushes, etc. Through EPCP, the potential positive anchors are selected as much as possible, which are called the first round of candidate positive samples, $C_{1}$, in this paper. However, it also brings a new problem, that is, how to select high-quality anchors from the many anchors in this center region. Therefore, this paper proposes the CLA for evaluating the quality of anchors in the next section.

### 3.2. Combined Loss of Anchor

**Figure 3:**
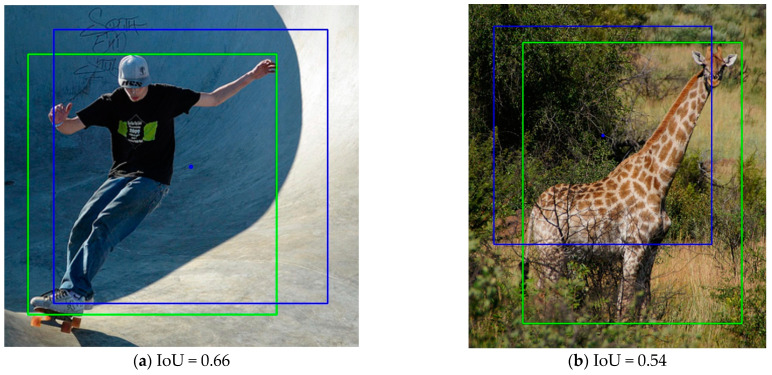
Examples of high IoUs, but poor intersection areas. The green box is the GT box. The blue point is one of the anchors, and the blue box is the anchor box of this point.

Using a fixed IoU threshold or other fixed hyperparameters as the basis for assignment often fails to assign the more suitable anchors to the GT box. For example, RetinaNet [1] uses a fixed IoU threshold and considers that the anchor boxes with larger IoUs with the GT box are positive samples. In Figure 3, the green box is the GT box, the blue box is one of the anchor boxes (only the anchor box with an aspect ratio of 1:1 is drawn), and the blue point in the center of the anchor box is the corresponding anchor point. The anchor point in Figure 3a is in the center region of the GT box, and the IoU between the anchor box and the GT box, which is 0.66, is also relatively large. This IoU value is higher than most anchor boxes. According to RetinaNet’s method, this anchor box will be assigned as a positive sample for training. However, most of the area where the anchor box intersects with the GT box is the background, and even the central area of the anchor box is mostly the background rather than the object, so it is difficult for the model to learn useful information from the content that is largely background noise. The prediction box obtained based on this anchor box is unlikely to have a high IoU with the GT box, and thus, ideal prediction results cannot be obtained. Therefore, although the IoU of this anchor box with the GT box is higher than most anchor boxes, it is not a suitable positive sample. From this example, it can be seen that the IoU between the anchor box and the GT box cannot be used as the only basis for evaluating the quality of the anchor box or anchor point.

ATSS [3] tiles only a square anchor box at each location and uses dynamic IoU as a threshold to distinguish between positive and negative samples. Although this approach can alleviate the problem caused by the fixed IoU threshold, there are still notable issues. On the one hand, since the IoU of the anchor boxes and the GT box is not the best metric to assess the quality of anchor boxes, using the sum of the mean and standard deviation of the IoU as the threshold is also not optimal; on the other hand, these tiled anchor boxes do not change in any way during the training process, so the IoUs of the GT box and anchor boxes do not change and the IoU threshold does not change with the training process, so the positive samples will not change and the model still cannot participate in the process of anchor assignment. In addition, both RetinaNet and ATSS do not consider the aspect ratio of the GT box. For example, the anchor box in Figure 3b has a high IoU with the GT box, but the intersection area is mostly background. Moreover, the length and width of the GT differ greatly, so it is difficult to satisfy all kinds of aspect ratios of GT boxes by only tiling one scale and aspect ratio of anchor boxes. Furthermore, tiling multiple scales and aspect ratios of anchor boxes not only requires setting the scales and aspect ratios of anchor boxes according to the dataset, but also greatly increases the computational effort.

Since the IoU between the best positive anchor box and the GT box is not necessarily higher than that of a negative anchor box, for the reasonableness of the sample assignment, the anchor needs a more appropriate evaluation metric to define whether the anchor is a positive sample or a negative sample. Furthermore, this metric needs to be relevant to the model to avoid the situation where the IoU of the anchor and the GT box is large during the assignment process, but the model still predicts poorly. The anchor assigned by this metric is not necessarily near the center of the GT box, and the corresponding IoU of the anchor box to the GT box is not necessarily high, but it can well represent the characteristics of the objects in the GT box, allowing the model to learn better. In summary, this paper proposes the CLA, which satisfies the above conditions, as shown in Equation (3):

(3) $$L_{\mathrm{CLA}}=L_{\mathrm{CLA}}^{cls}+\lambda_{1}L_{\mathrm{CLA}}^{reg}+\lambda_{2}L_{\mathrm{CLA}}^{dev}$$

where $L_{\mathrm{CLA}}^{cls}$, $L_{\mathrm{CLA}}^{reg}$, and $L_{\mathrm{CLA}}^{dev}$ are the classification loss, regression loss, and deviation loss of the prediction results of candidate anchors, respectively. $\lambda_{1}$ and $\lambda_{2}$ are hyperparameters to balance the weights of each loss. In the experiments of this paper, $\lambda_{1}=1.5$ and $\lambda_{2}=1$ are used.

The CLA takes into account both the classification quality and regression quality of the anchors, as well as the degree of deviation within the GT box. The classification loss and regression loss of anchors in CLA are similar to the anchor scores used in the PAA algorithm [4], which will be smaller for anchors that are suitable as positive anchors and, conversely, will be larger for negative anchors. In particular, the classification loss and regression loss will be larger for anchors that contain a large amount of background because it is almost impossible for the model to correctly predict the bounding box and the corresponding category based on the background without clues. In addition, since the center region where the $C_{1}$ are located is related to the stride S, this center region is large on the higher feature layers, leading to the selection of all anchors within some small and medium-sized objects on these feature layers. Therefore, this paper proposes the use of deviation loss. Anchors in $C_{1}$ at the edge of the GT box have different deviation loss from those at the center range of the GT box, but exactly which anchors are eventually selected as positive samples is determined by the CLA. The smaller the CLA, the better the prediction of the correct category and bounding box. The calculation of each loss is described below.

After the screening of the EPCP in the previous step, the corresponding candidate positive samples can be obtained on different feature layers, and the candidate positive samples in each layer together form the first round of candidate positive samples $C_{1}$. Suppose one of the anchors $a_{j}\in C_{1}$, whose coordinates are (x,y). $a_{j}$ obtains the prediction value $p_{x,y}=(p_{j}^{cls},p_{j}^{reg})$ after forward propagation of the mode, where $p_{j}^{cls}$ and $p_{j}^{reg}$ represent the classification vector and the coordinate vector of the regression box predicted by the model, respectively. If $a_{j}$ is assigned to the GT box, then $g_{i}=(x_{1}^{\left(i\right)},y_{1}^{\left(i\right)},x_{2}^{\left(i\right)},y_{2}^{\left(i\right)},c^{\left(i\right)})$, where $\left(x_{1}^{\left(i\right)},y_{1}^{\left(i\right)}\right)$ and $\left(x_{2}^{\left(i\right)},y_{2}^{\left(i\right)}\right)$ denote the coordinates of the vertices of the upper left and lower right corners of the GT box, and $c^{\left(i\right)}$ corresponds to the class of the objects in the GT box.

The classification loss of the anchors uses Focal Loss [1]. The vector $p_{j}^{cls}$ obtained from the forward propagation of the anchor $a_{j}$ is a vector with the dimensionality of the number of categories $N_{class}$, and its classification loss can be calculated as shown in Equation (4):

(4) $$L_{\mathrm{CLA}}^{cls}=\mathrm{FocalLoss}(p_{j}^{cls},g_{i})$$

The regression loss of the anchors uses GIoU loss [27]. The prediction box’s coordinate vector $p_{j}^{reg}$ obtained from the forward propagation of the anchor $a_{j}$ is a vector of 4 dimensions which can be expressed as $p_{j}^{reg}=(l_{j},t_{j},r_{j},b_{j})$, representing the position information of the prediction box relative to the anchor $a_{j}$ in the FCOS detector [2]. The values of the four components represent the distances from the anchor point to the left, top, right, and bottom boundaries of the prediction box, respectively. The regression loss can be calculated as shown in Equation (5):

(5) $$L_{\mathrm{CLA}}^{reg}=\mathrm{GIoULoss}(a_{j},p_{j}^{reg},g_{i}),$$

The deviation loss of the anchors is calculated by the center deviation. Suppose the distances from the anchor $a_{j}$ to the left, top, right, and bottom boundaries of the GT box $g_{i}$ are (l,t,r,b), respectively, and since the first round of screening ensures that the anchor $a_{j}$ is inside the GT box $g_{i}$, all 4 of these distances are positive. Based on these 4 distances, this paper defines the center deviation, denoted as dev, as shown in Equation (6). The smaller the absolute value of the difference |l−r|, the more this anchor is in the center of the GT box horizontally, and the same goes for the difference |t−b|. In addition, considering the different potential lengths and widths of the GT box, the absolute value of this difference is divided by the corresponding side length of the GT box and normalized to [0,1].

(6) $$dev=\frac{\left|l-r\right|}{l+r}+\frac{\left|t-b\right|}{t+b},$$

The deviation loss proposed in this paper is shown in Equation (7), and the deviation loss is set to 0 for anchors whose center deviation is within the threshold value, i.e., this anchor is considered to be within the acceptable range of deviation; the deviation loss is calculated for each anchor whose center deviation is greater than the threshold value, and the specific value is calculated by the dev.

(7) $$L_{\mathrm{CLA}}^{dev}=\left\{\begin{array}{cc} 0, & 0\leq dev\leq 1\\ dev-1, & 1<dev\leq 2 \end{array}\right.,$$

The above three losses together form the CLA, which takes into account the actual distribution of objects inside the GT box and the aspect ratio of the GT box. This metric is more reasonable for the anchor assignment of eccentric objects and objects with a large difference in length and width.

### 3.3. Dynamic Loss Threshold

After calculating the CLA of $C_{1}$, we need to divide the positive and negative samples in $C_{1}$. To further divide the positive and negative samples, a fixed number of positive samples is directly used in LLA [28] without considering the specific value of anchor loss, and only the k anchors with smaller losses are selected as the positive samples. Although this approach does not require additional calculations, it introduces hyperparameters and cannot use the specific value of the loss to judge the number of positive samples. Moreover, the number of positive samples is not necessarily the same for objects of different sizes. A complex GMM is used in PAA [4] to cluster the candidate positive samples according to the value of the anchor score (calculated by the anchor loss) into two categories of positive and negative samples. PAA greatly reduces the training speed of the model; for each GT box, it needs to be re-iterated once, and this iteration needs to be performed on the CPU.

To solve the above problems and better use anchor loss to dynamically divide the positive and negative samples without using additional models, this paper proposes a simpler and more effective division method, called the DLT. After calculating the CLA of $C_{1}$, the process of using DLT is as follows:

1. Select k anchors with smaller CLAs in each feature layer to obtain the second round of candidate positive samples $C_{2}$.
2. Select m anchors with smaller CLAs in $C_{2}$ as the third round candidate positive samples $C_{3}$, and calculate the mean value $t_{g}$ of the CLAs of these m candidate positive samples.
3. The anchors in $C_{3}$ with CLAs lower than $t_{g}$ are taken as positive samples $A_{P}$, and the rest are negative samples $A_{N}$.

Since the LADA algorithm does not focus on which feature layer is more suitable for predicting the current GT box in the first round using EPCP screening, $C_{1}$ may come from all feature layers, and some feature layers are not suitable for predicting the GT box at the current scale. To find suitable positive samples, DLT in LADA first selects k anchors in each feature layer to form the second round of candidate positive samples $C_{2}$. However, there are some candidate anchors on feature layers that are not suitable for predicting the current GT box (e.g., anchors on the lowest feature layer are not suitable as their candidate anchors were placed when the GT box was larger), these poorer candidate anchors have larger losses and are not suitable as positive samples, and there is no need to continue to retain these anchors. However, PAA retains these poorer anchors and clusters them as well. Since these poor candidate anchors have larger losses, they are also assigned as negative samples after clustering. Therefore, anchors in $C_{2}$ all participating in clustering is not the most appropriate approach, because some anchors with larger losses and low rankings will hardly be positive samples. In addition, the cost of GMM iteration is large, and the same process has to be performed for each GT box. Furthermore, an image often has more than one GT box, which leads to the training time being greatly prolonged.

Therefore, the DLT proposed in this paper does further screening on $C_{2}$ before dividing it. The experimental part of this paper demonstrates that compared with the PAA algorithm, the LADA algorithm reduces the training time by about 26.8%.

### 3.4. Algorithm Realization

As shown in Algorithm 1, the process of positive and negative sample assignment by LADA is described. A preliminary matching is performed before the assignment, referring to the practice in PAA [4] that assigns the anchors to the GT box with the largest IoU. Meanwhile, the anchor-free detector refers to the practice in ATSS [3] that gives each anchor a flat 8×S anchor box before the preliminary IoU matching. When an anchor is assigned to more than one GT box, the GT box with the largest IoU of the anchor will be selected. The IoU threshold here is consistent with that in PAA, set to 0.1. The anchors containing too much background are omitted, and the most original positive sample candidate set is constructed. The anchors both in the GT box and the center region of EPCP are the first round of candidate positive samples $C_{1}$. Then, the CLA of $C_{1}$ is calculated, and k anchors with smaller CLAs are selected in each layer as the second round of candidate positive samples $C_{2}$. Finally, m anchors with smaller CLAs are selected as the third candidate positive samples $C_{3}$. The mean CLA value of $C_{3}$ is calculated as the DLT to distinguish the positive and negative anchors, so the positive anchors $A_{P}$ and negative samples $A_{N}$ are obtained.
**Algorithm 1** Lightweight Anchor Dynamic Assignment (LADA)Input: G, A, $A_{i}$, F, K, M. G is a set of GT boxes, A is a set of anchors, $A_{i}$ is a set of anchors from $i_{th}$ pyramid level, F is the number of pyramid levels, k is the number of the second round of candidate positive samples $C_{2}$ for each pyramid, m is the number of the third round of candidate positive samples $C_{3}$. Output: $A_{P},A_{N}$. $\;A_{P}$ is a set of positive samples, $A_{N}$ is a set of negative samples.
1.$A_{P}\leftarrow Ø,A_{N}\leftarrow Ø$4.for g∈G do ⊳ Iterate through each GT box in the current image5.$L\leftarrow Ø,C_{j}\leftarrow Ø,j=1,2,3$⊳ $C_{j}$ is the $j_{th}$ round of candidate positive samples6.$C_{1}\leftarrow \mathrm{EPCP}(A,g)$⊳ Select the anchors using EPCP7.for i=1,2,…,F do8.$L_{i}\leftarrow \mathrm{CLA}(g,C_{1}\cap A_{i})$⊳ Calculate the CLA of this layer from Equation (3)9.$L\leftarrow L\cup L_{i}$⊳ The CLAs of each layer of anchors together form L10.$C_{2}\leftarrow C_{2}\cup k^{th}\mathrm{MinCLA}(L_{i},C_{1})$⊳ Select k anchors with smaller CLAs as $C_{2}$11.end for12.$C_{3}\leftarrow m^{th}\mathrm{MinCLA}(L,C_{2})$⊳ Select m anchors with smaller CLAs13.$t_{g}=\mathrm{DLT}(C_{3})$⊳ Calculate the mean of CLA as DLT14.$\mathrm{for}\;c\in C_{3}$ do15.if $L(c)<t_{g}$ then16.$A_{P}=A_{P}\cup c$⊳ Anchors with CLAs lower than $t_{g}$ are positive anchors17.end if18.end for19.end for20.$A_{N}\leftarrow A-A_{P}$21.return $A_{P},A_{N}$

**Figure 4:**
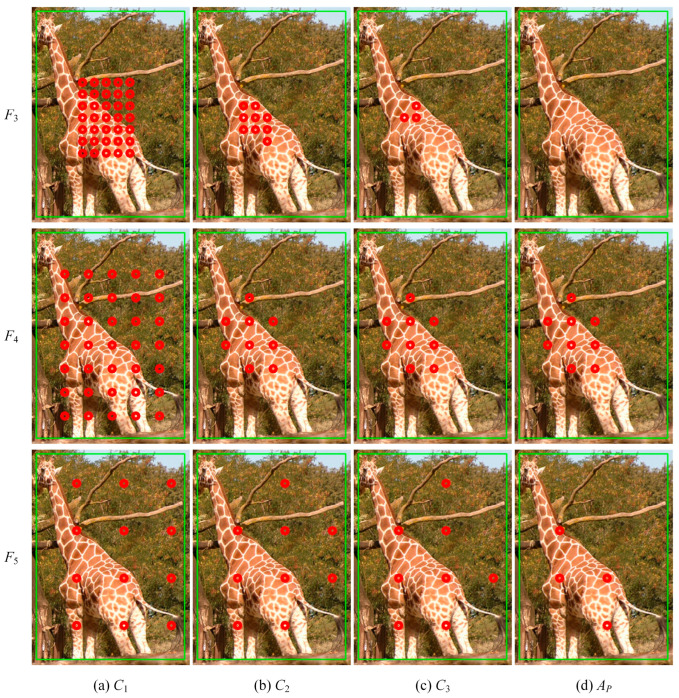
Diagram of the LADA implementation. The green box is the GT box and the red dots are positive anchors of each stage.

The assignment process is schematically shown in Figure 4. To illustrate the assignment of different feature layers, three of them, $F_{3}$, $F_{4}$ and $F_{5}$, are selected and the positions of the assignment results of these feature layers on the original figure are drawn. Anchors in Figure 4a are the first round of candidate positive samples that satisfied EPCP. Anchors in Figure 4b are candidate samples with smaller CLAs in each layer. Anchors in Figure 4c are candidate samples with smaller CLAs in all layers, and it can be seen that there are almost no anchors retained in the feature layer $F_{3}$, which is not suitable for prediction. Anchors in Figure 4d are positive samples with CLAs smaller than the threshold, and the positive samples are only distributed in $F_{4}$ and $F_{5}$.

**Figure 5:**
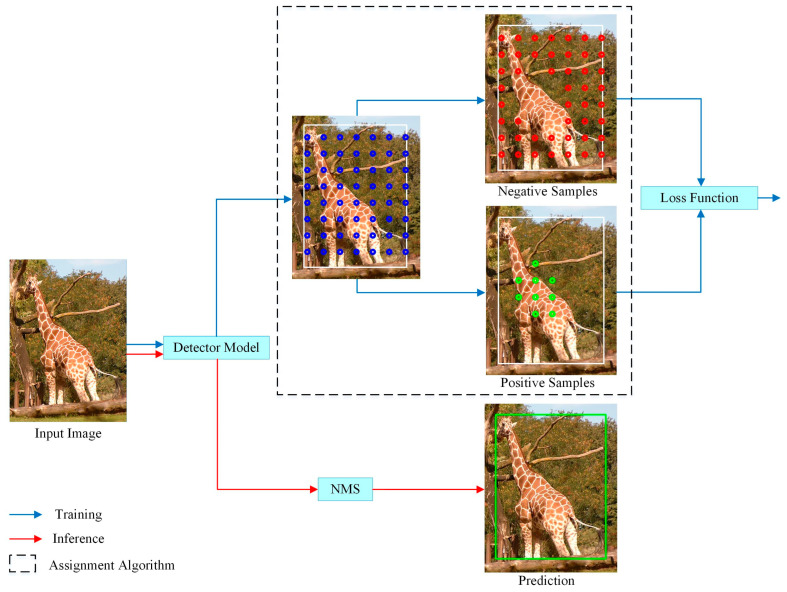
Assignment algorithm in the training process. In the training part, the white box is the GT box, and the blue dots are all candidate anchors. By using assignment algorithm, candidate anchors are divided into positive samples (green dots) and negative samples (red dots). In the inference part, the prediction result (green box) is obtained after NMS without assignment algorithm.

The LADA algorithm proposed in this paper is used in the training phase, as shown in Figure 5. In the training process, the positive and negative samples are determined by LADA, and then the losses, such as classification and regression, of the model prediction results are calculated and back-propagated to update the parameters of the detection model. The inference process, on the other hand, does not require the involvement of the LADA algorithm and only requires Non-Maximum Suppression (NMS) as post-processing of the model output vectors to obtain the detection results. Since the LADA algorithm only changes the positive and negative sample assignment results, it does not change the model structure.

## 4. Experimental

### 4.1. Dataset and Evaluation Protocols

All experiments in this paper were conducted on the challenging MS COCO [29] dataset, which consists of 80 categories. Images in trainval35k (approximately 118 k images) were used as the training set and images in minival (5 k images) were used as the validation set according to the settings in FCOS [2], ATSS [3], and PAA [4].

Average Precision (AP) for a common individual category is defined as the average value of precision values on the PR curve. mAP (mean Average Precision) is defined as the average value of AP for each category under a certain IoU threshold $t_{IoU}$, as shown in Equation (8):

(8) $$\mathrm{mAP}(t_{IoU})=\frac{\sum_{i=1}^{N_{class}}{\left(\mathrm{AP}\right)}_{i}}{N_{class}},$$

where $N_{class}$ is the number of categories. The evaluation metrics used in the experiments of this paper are consistent with [2,3,4], using mainstream COCO evaluation metrics, mainly including AP, AP50, AP75, APs, APm, and APl, where AP is the main evaluation metric, using the mean value of mAP at different IoU thresholds. The value of mAP is calculated every 0.05 in the interval of IoU from 0.50 to 0.95, and the average value of all results was taken as the final result. Therefore, the AP in COCO evaluation metrics is more stringent and is no longer the common AP of a single category. The AP values mentioned in the experimental data in this paper are all calculated in this way, which is the most commonly used evaluation metric in the COCO dataset. AP50 and AP75 denote the mAP values when the IoU threshold is 0.50 and 0.75, respectively, that is, AP50 for $\mathrm{mAP}(t_{IoU}=0.5)$ and AP75 for $\mathrm{mAP}(t_{IoU}=0.75)$. APs, APm, and APl represent the AP of small, medium, and large targets, respectively.

### 4.2. Implementation Details

#### 4.2.1. Detectors

Similar to ATSS and PAA, the LADA proposed in this paper is applicable to both anchor-based and anchor-free detection models. Typical anchor-based and anchor-free detectors are RetinaNet [1] and FCOS [2], respectively. The experiments in this paper were mainly based on the FCOS detector, and relevant experiments were also conducted on the RetinaNet detector to demonstrate the generality of the LADA algorithm. The experiments used the original RetinaNet detector and the FCOS detector with ResNet-50 backbone as baselines, where RetinaNet uses IoU as the basis for anchor assignment, while FCOS uses scale constraints and spatial constraints with center sampling as the basis for anchor assignment. In addition, the experimental results are compared with other sample assignment algorithms to illustrate the effectiveness of the LADA.

The FCOS used in the experiments remains the same as [21] with some added improvements, i.e., adding GroupNorm [30] in the heads, using GIoU loss as the regression loss function, limiting the positive samples in the GT box, adding a trainable scalar for each feature layer, and adding an auxiliary branch in parallel with the regression branch. However, the auxiliary branch is no longer the centerness branch, but the IoU branch. The RetinaNet used in the experiments follows the setup in ATSS [3]. In addition to the sample assignment method, the differences are corrected by adding the improvements used in the FCOS detector to the RetinaNet detector. In addition, RetinaNet has only one anchor box pre-defined at each location. If not otherwise specified, the above settings are used for RetinaNet and FCOS mentioned in this paper.

**Figure 6:**
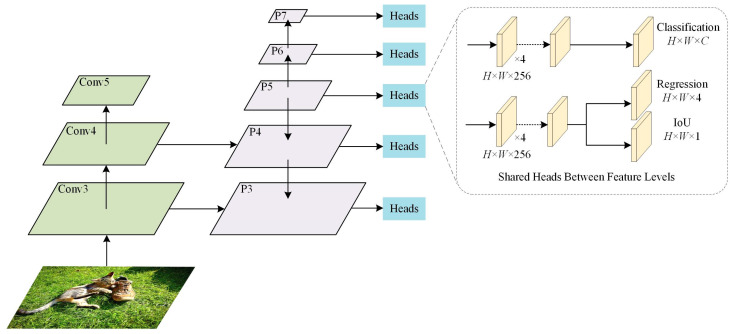
The detector architecture used in the experiments.

The structure of the detector is shown in Figure 6; it is similar to that in FCOS and ATSS, but the auxiliary branch is an IoU branch, and this structure is the same as the one used in PAA.

To ensure the same conditions for the experiments, the following modifications were made to PAA [4] in the experiments of this paper:

1. Using a 1× training period to train 12 epochs on trainval35k of MS COCO instead of the 1.5× training period mentioned in PAA.
2. Not additionally using the score voting method mentioned in PAA after NMS.
3. The PAA in [4] is only applied to the RetinaNet detector in the above setup, and the experiments in this paper also use the PAA in FCOS for comparison.

All settings remain the same except for the assignment algorithm.

#### 4.2.2. Training and Inference Details

The experimental environment is a setup with an Intel Core i9-10900K CPU@3.70 GHz, NVIDIA GeForce RTX 3090, 64 GB of RAM, and the operating system is Ubuntu 18.04.

The model uses ResNet-50 [31] pre-trained by ImageNet [32] and a five-layer feature pyramid structure as the backbone network, both with a training period of 1×. According to the linear strategy, the batch size of training was adjusted to eight for 180 k iterations (i.e., 12 epochs). During the training process, the size of the input image was adjusted so that the short edge was 800 and the long edge was less than or equal to 1333. The whole network was trained using the Stochastic Gradient Descent (SGD) algorithm with a momentum of 0.9 and a weight decay of 0.0001. The initial learning rate was set to 0.005 and decayed to one-tenth of the original at iterations up to 120 k and 160 k, respectively. Multi-scale training was not used during the training. If not specified, the hyperparameters k in the experiments were set to nine, consistent with those in ATSS and PAA. The hyperparameters m were set to 20.

In inference, the input images were first resized using the same method as in the training phase, and the predicted categories and corresponding bounding boxes were obtained by forward propagation through the entire network. Then, a threshold of 0.05 was set for the score of the corresponding category in the classification vector, the prediction boxes containing a large amount of background were filtered out, and the first 1000 high-confidence detections of each feature layer were output. Finally, the IoU threshold was set to 0.6 per class for NMS to generate the first 100 detections. The inference was set to be consistent with ATSS, and no additional means to enhance model performance, such as multi-scale testing and score voting, were not used. No additional means to enhance performance other than NMS were used in post-processing.

#### 4.2.3. Loss Functions

The loss function for training consisted of three parts, as shown in Equation (9):

(9) $$L=\sum_{\left(x,y\right)}\frac{1}{N_{pos}}L_{cls}(p_{x,y},g_{x,y})+\frac{1}{N_{pos}}1_{\left\{c_{x,y}^{*}>0\right\}}[\alpha_{1}L_{reg}(p_{x,y},g_{x,y})+\alpha_{2}L_{aux}(p_{x,y},g_{x,y})],$$

where $N_{pos}$ is the number of positive samples, (x,y) is the coordinates of the anchor, $p_{x,y}$ is the predicted value of the anchor, and $g_{x,y}$ is the true label corresponding to the anchor. $L_{cls}$ is the loss of the classification branch, using Focal Loss [1]; $L_{reg}$ is the loss of the regression branch, using GIoU Loss [27]; and $L_{aux}$ is the loss of the auxiliary branch, using BCE loss. In ATSS and FCOS, the auxiliary branch is the centerness branch. In PAA and this paper, the auxiliary branch is the IoU branch and $L_{aux}$ is the loss of the predicted IoU and true IoU. $\alpha_{1}$ and $\alpha_{2}$ are the weights used to balance the loss, and the weights used in this paper are consistent with ATSS, that is, $\alpha_{1}=2$ and $\alpha_{2}=1$. $1_{\left\{c_{x,y}^{*}>0\right\}}^{}$ is the indicator function when $c_{x,y}^{*}>0$; that is, when the anchor with coordinates (x,y) is a positive sample, $1_{\left\{c_{x,y}^{*}>0\right\}}^{}$ = 1, otherwise $1_{\left\{c_{x,y}^{*}>0\right\}}^{}$ = 0.

### 4.3. Experimental Results and Analysis

#### 4.3.1. Effect of Each Individual Component

The LADA algorithm has three main components, which are the EPCP, CLA that contains the deviation loss, and DLT. To explore the effect of each component on FCOS, experiments were designed, and the results are shown in Table 1. When the EPCP is not used, the normal center prior is used by default, i.e., the positive sample anchors are restricted to be within the center region of the square. When the CLA is not used, the loss of anchors includes only classification loss (using Focal Loss) and regression loss (using GIoU Loss), excluding the deviation loss proposed in this paper. When the DLT is not used, the number of positive samples is fixed to 10 to divide the positive and negative samples.

As can be seen from the results in Table 1, using DLT to distinguish between positive and negative samples (40.26%) gained 0.3% over using a fixed number of positive samples (39.96%), indicating that the number of positive samples required varies between different GT boxes, and DLT can better calculate the actual threshold to determine the number of positive samples in different situations. Using CLA that includes deviation loss or adding EPCP as the center prior approach on top of this, the AP can be improved by 0.08% and 0.1%, respectively, demonstrating that deviation loss can be used to make a further assessment of anchor quality on top of the original loss, and EPCP can better retain suitable candidate anchors than CP, thus improving the AP performance. When we use both EPCP and CLA, the AP of the model improves from 40.26% to 40.46%, which is better than using EPCP alone (40.34%) and CLA alone (40.36%), indicating that these two components work orthogonally, and using either of them can give a boost to the AP of the model. Overall, LADA improves the AP from 39.96% to 40.46%, and each component of LADA provides a different degree of performance improvement.

#### 4.3.2. Effect of Center Prior

(1) Effect of EPCP

To illustrate the role of the EPCP, the CLA and the DLT in the LADA algorithm are applied to the FCOS detector by using the CLA as the anchor quality assessment and the DLT as the positive and negative sample division method, modifying only the center prior, as shown in Table 2. The ordinary CP and the EPCP are compared, respectively, as well as the impact on performance with different hyperparameters r. It can be seen that the model performs best when the hyperparameter r=2.5. In addition, the model is not sensitive to the parameter r. The AP performance of FCOS can be boosted from 40.35% to 40.46% by using the EPCP. As the EPCP is primarily a correction for objects with a large difference in length and width, further experiments will be conducted later to compare the AP values of these objects and demonstrate the usefulness of the EPCP.

We also applied the LADA with EPCP to the RetinaNet detector in the above manner, and the results are shown in Table 3. After applying the EPCP to RetinaNet, the AP performance improved from 40.33% to 40.41%. A slight decrease in APl was found after using EPCP, but AP is the main evaluation metric and better reflects the actual performance of the model.

(2) Comparison of various categories of objects

Since the EPCP is mainly corrected for objects with a large difference in length and width, to better illustrate the effect of the EPCP on these objects, this paper selects some of the 80 categories of the COCO dataset for comparison, such as bicycle, giraffe, tennis racket, etc. In this paper, these categories of objects are referred to as “slender objects”. The distribution of more meaningful anchors is not all in the center square region for these objects. Table 4 compares the AP performance of different algorithms on the COCO minival set for slender objects. Among them, FCOS with CP uses the centerness branch and the rest uses the IoU branch. For these classes with different aspect ratios, the AP can be improved by 1% or more after using the EPCP. This indicates that for categories with a large difference in length and width, changing the center region when selecting candidate samples using the EPCP can effectively improve recognition of these objects and enhance overall detection performance.

(3) Candidate positive samples after EPCP

**Figure 7:**
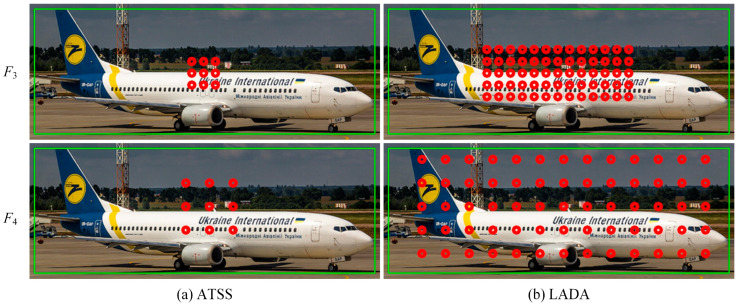
Comparison of the first round of candidate positive samples. The green box is the GT box, and the red dots are candidate positive anchors.

For slender objects, EPCP selects anchors according to their length and width when selecting the first round of candidate positive samples, while ATSS uses L2 distance as the basis for selecting the first round of candidate positive samples without considering the GT box’s aspect ratio. Therefore, ATSS is selected as the comparison in the experiment. The candidate positive samples for different center priors are shown in Figure 7. Figure 7a,b show the results of ATSS and LADA, respectively. Row 1 and row 2 are the candidate positive samples for the feature layers $F_{3}$ and $F_{4}$, respectively. The GT box labeled by the aircraft in the figure is not square, and ATSS selects the nearest anchors to the center of the GT box as the first round of candidate positive samples, so these candidate positive samples are distributed in a small central range. The LADA proposed in this paper uses an EPCP and selects more candidate samples in the first round, as shown in Figure 7b, to keep the anchors that better represent the object position as much as possible, instead of only keeping the anchors in the center of the square or circle. Since the number of candidate samples is increased, some of the candidate positive samples will contain a large amount of noise, and these samples will be further filtered. Therefore, the EPCP is mainly to retain the anchors that are meaningful as much as possible, and the background will be screened in the next round. If the ATSS method is used, as shown in Figure 7a, not many anchors containing useful information are obtained in the candidate samples. However, with the EPCP, as shown in Figure 7b, almost all the anchors that can represent the object features are retained; some of them contain a large amount of background, but the high-quality anchors are also retained. The background anchors will be further filtered by calculating the CLA and DLT, so the background anchors do not affect the model due to being classified as positive samples.

(4) EPCP applied to FCOS without CLA and DLT

Since the proposed EPCP is an improvement over the ordinary center prior, this paper designed experiments to modify the square center region obtained from CP in FCOS to the center region obtained from EPCP. The FCOS follows the settings in [21], using a centerness branch, and the settings are the same except for the difference in the center prior approach. It should be noted that since EPCP increases the area of the candidate region, it leads to the introduction of some noise, while FCOS uses almost all anchor points in the candidate region directly as positive samples. Therefore, a simple correction is made to EPCP in the experiment. This is accomplished as follows: after calculating the aspect of the GT box, the area of the central region $S_{r}$ obtained according to the CP is calculated, and then a new central region is generated according to the aspect ratio, whose area is still $S_{r}$, and the aspect ratio is the same as that of GT box.

**Figure 8:**
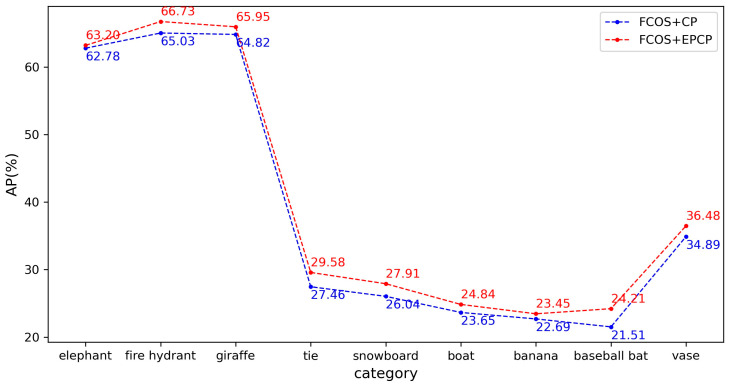
Different center prior type performance on the COCO minival set.

The experimental results are shown in Table 5, which shows that the performance of FCOS is slightly improved after adding EPCP. To better compare the improvement brought by EPCP, we selected the AP performance of slender objects, as shown in Figure 8. The AP values of all these objects were improved by varying degrees up to 2.7% by FCOS with the use of EPCP.

#### 4.3.3. Effect of Anchor Score

To achieve the assignment of positive and negative samples, an evaluation metric is needed to measure the quality of anchors, called anchor quality assessment. In Faster R-CNN, RetinaNet, and ATSS, anchor quality assessment uses the IoU of the anchors and the GT box. To demonstrate that loss of anchor (LA) is more suitable for anchor quality assessment than IoU, Table 6 compares the performance of the model when IoU and LA are used as anchor quality assessment metrics. IoU is the method used in ATSS, which uses the statistical properties of the IoU of the anchors and the GT box to divide the anchors; LA is used as the other anchor quality assessment metric and consists of classification loss (using Focal Loss) and regression loss (using GIoU Loss), but does not include the deviation loss proposed in this paper, does not use any center prior, and only restricts the samples to be within the GT box. Both models have the same structure of FCOS with the IoU branch as an auxiliary branch. It can be seen that even without using any center prior, using LA improves the AP by about 0.37% over IoU, indicating that loss of anchor is more representative of the quality of the anchor, and using LA instead of IoU is feasible.

Now, we have verified that LA can be used as a metric for anchor quality assessment instead of IoU. Then, it is possible to further investigate whether the improvement of LA can further improve the performance of the model. In this paper, we propose a deviation loss, which is further modified based on the original LA to form a new CLA. Since the center prior has a large impact on the performance of the model, we use the EPCP as the center prior in the following experiments, use the DLT to divide the positive and negative samples, and only change the composition of the losses in the anchor quality assessment to study the role of different losses.

The experimental results are shown in Table 7. Using GIoU instead of IoU as the regression loss can improve AP performance from 40.33% to 40.36%, and adding deviation loss on top of this can further improve AP performance to 40.46%.

#### 4.3.4. Effect of Sample Division

(1) Effect of dynamic sample division

To illustrate the role of the dynamic number of positive samples $N_{pos}$, this paper designed the following experiment on the FCOS detector: First, the LA, which contains only the classification loss and regression loss, is calculated. The first way to divide samples is to fix the number of positive samples; that is, according to the anchor loss from low to high, the first $N_{pos}$ candidate anchors with smaller LA values are selected as positive samples, which leads to the same number of positive samples for all GT boxes in all images and keeps the number constant during training. PAA uses the GMM to dynamically divide the anchors into positive and negative samples through clustering according to the LA. PAA* in this paper means that the GMM is not used to determine the number of positive samples, so PAA can be expressed as “PAA* + GMM”. The method proposed in this paper is “PAA* + DLT”, which means that the DLT is used to replace the GMM in the original PAA to determine the dynamic number of positive samples.

The experimental results are shown in Table 8. If the number of positive samples is determined using a dynamic approach, either using the GMM or using the DLT proposed in this paper, a better AP performance than a fixed number of positive samples can be obtained. In addition, the AP performances of the GMM and DLT approaches under the COCO minival set were 40.22% and 40.26%, respectively, and the AP using the PAA* + DLT approach was 0.04% different than that of the PAA. This indicates that the DLT in this paper achieves a similar effect as GMM. Later experiments will demonstrate that the DLT proposed in this paper greatly reduces the training cost compared to GMM in PAA.

(2) Number of positive samples in the training process

**Figure 9:**
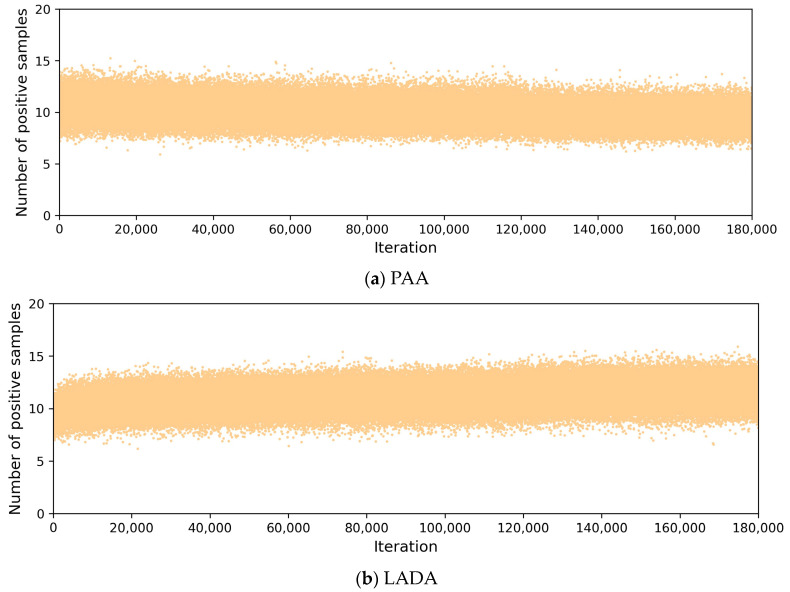
Comparison of the number of positive samples during training.

To compare with PAA, which divides samples dynamically, Figure 9 plots the number of positive samples in the training processes of PAA and LADA. The number of positive samples per iteration in Figure 9 means the average of the number of positive samples of all GT boxes in all images in one iteration, totaling 180k iterations, and the number of images in one iteration is equal to the batch size. In Figure 9a, the positive samples of PAA were obtained by iterating the GMM, and the number changed dynamically. From Figure 9b, we can see that the number of positive samples selected by DLT in LADA for each iteration is also not fixed, but determined according to the CLA, and the number of positive samples is mainly concentrated in the range of 8 to 14, showing better adaptability. Both LADA and PAA can determine the number of positive samples dynamically according to the loss of anchor.

In addition, experiments are conducted, with the results shown in Table 9, for different values of m in DLT. When m is small, there are fewer candidate samples with very small CLA values, which will lead to a very low loss threshold and fewer samples with CLA values less than this threshold. When m is larger, there are too many low-quality samples participating in the calculation of the loss mean, making the loss threshold larger, and some anchors with larger CLA values are also selected as positive samples. From the experimental results, it can be seen that the AP performance of the hyperparameter m is best when it takes the value of 20, while increasing or decreasing the value of m will slightly decrease the performance. In general, DLT is not sensitive to the hyperparameter m.

(3) Dynamic positive samples during training

**Figure 10:**
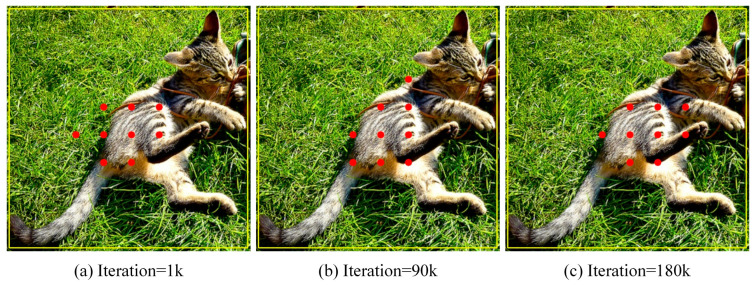
Positive samples at each stage of the training process. The red dots are positive anchors of each training stage.

To demonstrate that the positive samples selected during the training process change with the training state of the model, three stages of the training process were selected in an experiment, as shown in Figure 10, which shows the positive samples finally assigned at the early training stage (iteration = 1 k), the middle training stage (iteration = 90 k), and the late training stage (iteration = 180 k). At the early stage of training, as shown in Figure 10a, the positive samples are mainly concentrated in the center region of the GT box because of EPCP, and the positive samples do not fully reflect the distribution of the object; some positive samples are distributed at the junction of the object and the background, and even some positive samples are located in the region that is basically the background. As the training proceeds, the recognition ability of the model improves, and the difference in the CLA of different quality anchors becomes obvious. When calculating the CLA, the CLA of anchors is more representative of the good or bad quality of anchors, which further provides suitable positive samples for training. Therefore, by the middle of training, as shown in Figure 10b, the anchors containing a lot of noise become fewer in the selected positive samples, and the distribution of anchors becomes more reasonable. By the later stage of training, as shown in Figure 10c, the selected positive samples are better optimized and basically do not contain too much background noise. Even though some anchors are in the center region, they are not selected because these anchors contain a lot of background, but the anchors that better represent the object features are selected as positive samples. So, the selected anchors are not necessarily all just in the center region.

#### 4.3.5. Comparison with Other Methods

(1) Comparison of positive samples

**Figure 11:**
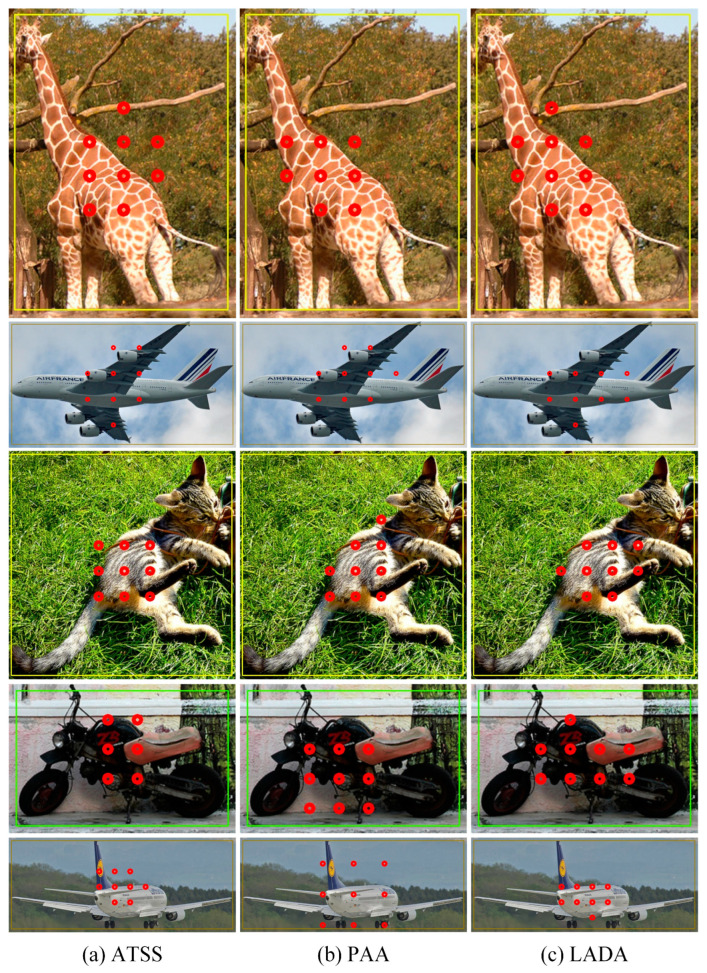
Comparison of anchor assigning results. The rectangles boxes are the GT boxes, and the red dots represent the final positive anchors.

For some objects that are not exactly in the center of the GT box, if the ATSS algorithm is used for the assignment, as shown in Figure 11a, positive anchors are almost all gathered in the center region of the GT box. Anchors in the background are also selected when there is a background in the center region, so the anchors selected by ATSS do not represent the object well and are less adaptive. The PAA algorithm uses the classification loss and regression loss of the anchors to mitigate the effect brought by the background region in the center to some extent, as shown in Figure 11b. The LADA algorithm proposed in this paper can make full use of the aspect ratio of the GT box during the training process, and calculate the CLA to obtain more suitable anchors based on the prediction of each anchor. As shown in Figure 11c, the selected positive anchors are not all in the center region, but fit with the actual distribution of the objects. For the anchors that are in the center region, but contain a large amount of background, they will not become positive samples after filtering. In addition, due to the use of the EPCP and the deviation loss, the selected positive samples are more consistent with the real object distribution, contain less background, and are assigned in a simpler way with shorter training time compared to PAA; the results of the training time comparison will be illustrated later.

(2) Comparison of training costs

Since the LADA algorithm only redefines the positive and negative samples and does not change the model structure, it does not lead to more training parameters for the model or result in additional overhead. We define the average hourly boosted AP value during training, denoted as $h_{\mathrm{AP}}$, as shown in Equation (10):

(10) $$h_{\mathrm{AP}}=\frac{\mathrm{AP}}{T},$$

where T is the training time in hours. A higher $h_{\mathrm{AP}}$ indicates a higher boosted AP per unit of time and a more efficient algorithm.

In Table 10, we record the GFLOPs, parameters, training time (in hours), and $h_{\mathrm{AP}}$ of the different anchor assignment algorithms for the FCOS detector with the same training settings of 1× schedule (12 epochs). In addition, we also record the performance of the RetinaNet detector. The settings for RetinaNet in the table remain the same as in [1], with nine anchor boxes tiled at each anchor. The model of RetinaNet is more computationally intensive due to the large number of anchor boxes required, which generate more parameters related to the anchor boxes and require the calculation of more IoU values between the GT box and anchor boxes. FCOS does not use anchor boxes and the number of parameters and FLOPs are smaller than RetinaNet. ATSS, PAA, and LADA all use the same FCOS detector, so the number of parameters and FLOPs are the same. FPS in Table 10 is measured on the same machine with a single GeForce RTX 3090 GPU using a batch size of one. Since the anchor assignment algorithm does not affect the inference process, the FPS at inference is similar. LADA maintains a higher detection speed and accuracy without increasing the model’s parameters and FLOPs.

It can also be seen from Table 10 that the $h_{\mathrm{AP}}$ of PAA is the lowest among all the methods, indicating that the GMM in PAA algorithm affects the training time substantially. Compared with PAA, LADA reduces the training time by about 26.8% and achieves better anchor assignment results with a 0.24% improvement in AP over PAA. Since PAA requires iterating the GMM on the CPU, it leads to a significant increase in training time. In addition, LADA only increases the training time by about 3 h over FCOS and ATSS. The approximate increase in training time compared to ATSS is 12.0%, but the AP value is boosted by 0.76%. The extra training time of LADA is mainly due to the demand of calculating the CLA values of prediction results from the true value in advance, but this calculation is performed on the GPU and does not significantly slow down the training process. In addition, RetinaNet tiles a large number of anchor boxes and the computational load of the IoU during training is high, which results in slower training speed than FCOS and does not bring additional boosts for AP. Therefore, corresponding to edge devices, tiling a large number of anchor boxes is not an appropriate choice.

The anchor assignment algorithm affects the training time because the detection models are set up in the same way, except for RetinaNet, and they have different training times. For FCOS, the center region needs to be calculated during training and the maximum distance from each anchor in the center region to the GT box is calculated. ATSS needs to calculate the L2 distance for each anchor and calculate the IoU values of the corresponding anchor boxes of the candidate anchors to the GT box, as well as the mean and standard deviation of the IoU values. Both PAA and LADA need to perform initial matching using IoU, and LADA needs to calculate the center region using EPCP after matching. Then, PAA and LADA calculate the classification loss and regression loss of the anchors, and LADA needs to additionally calculate the deviation loss. Finally, PAA needs to iterate through each GT box in an image and perform GMM iterations on the candidate anchors of each GT box to achieve clustering. Since there are often multiple GT boxes in an image, PAA needs iterate over each GT box again to convergence or to the maximum number of iterations during training, resulting in a much longer training time. LADA, on the other hand, uses DLT and only calculates the mean of the candidate anchors’ CLA values to separate positive and negative samples, with minimal computational cost. So, LADA is much faster to train than PAA in theory. The results in Table 10 also demonstrate that LADA reduces training time by about 26.8% compared to PAA.

(3) Comparison of AP performance

Table 11 compares the performance of using different anchor assignment algorithms on the FCOS detector. CP represents the center sampling based on scale constraints and spatial constraints used in FOCS [21]. Compared to FCOS and ATSS, which use the centerness branch, LADA boosted the AP by about 1.76% and 1.27%, respectively. When the model structure is exactly the same, LADA boosts 0.76% in AP, 0.13% in AP50, 0.89% in AP75, 0.33% in APs, 1.08% in APm, and 3.28% in APl compared to ATSS. Compared with PAA, it boosts about 0.24% in AP, and LADA achieves a simpler and more efficient process of sample assignment. The above experimental results illustrate the importance of positive and negative sample assignment to the model during training and the role of LADA.

In addition, related experiments were conducted on RetinaNet in this paper. Table 12 compares the performance on the RetinaNet detector using different anchor assignment algorithms. TH(IoU) represents RetinaNet [1] using fixed IoU as the basis for positive and negative sample assignment. When the model structures are identical, LADA improves AP over ATSS by 0.74%, AP50 by 0.35%, AP75 by 1.06%, APs by 0.81%, APm by 1.15%, and APl by 1.82%. Compared to PAA, it improves AP by about 0.21%. Thus, for both anchor-free and anchor-based detectors, LADA outperformed other anchor assignment algorithms, such as ATSS, PAA, and so on.

(4) Detector Result

**Figure 12:**
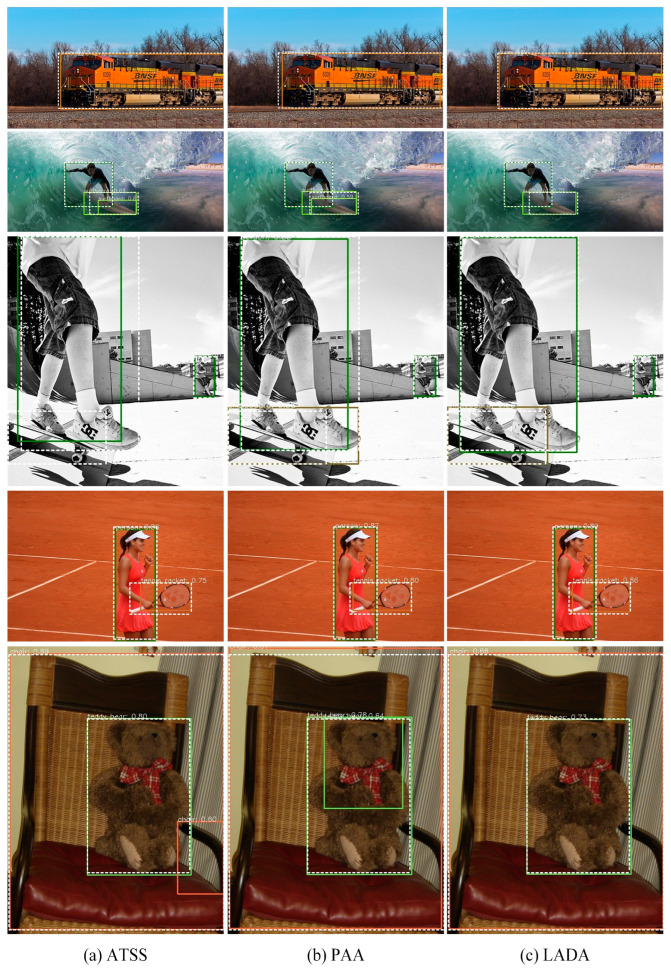
Comparison of prediction results of FCOS on the COCO minival set. The white dashed boxes are the GT box and other solid boxes are prediction boxes of each object.

Figure 12 plots the performance of different assignment algorithms on the FCOS detector for some images on the COCO minival set. The white dashed boxes are the GT boxes and the solid boxes represent the predicted boxes. It can be seen that for slender objects such as trains and people, ATSS and PAA do not consider the aspect ratio of the GT boxes, and some of the boundaries of the predicted boxes deviate significantly from the GT boxes’ boundaries. The predicted boxes of LADA are more accurate and have a higher overlap with the GT boxes. In addition, among the selected images, LADA has no redundant prediction boxes after NMS, and the false detection rate is smaller; meanwhile, ATSS and PAA may have redundant prediction boxes after NMS for some slender objects.

## 5. Conclusions

In this paper, we propose a new anchor assignment algorithm called LADA, which makes use of the aspect ratio of the GT box and selects the anchors that better represent the object features as the candidate positive samples as much as possible. It considers the actual distribution of objects in the GT box, makes the sample assignment more reasonable for eccentric objects and slender objects, solves the problems of poor adaptability and difficulty in selecting the better positive samples when assigning positive and negative samples based on IoU, and does not excessively increase the training cost. In this paper, we apply this algorithm to FCOS, an anchor-free detector, and RetinaNet, an anchor-based detector. Experiments show that the algorithm can effectively improve the performance of these two detectors on the MS COCO minival set and prove the effectiveness of LADA. The improvement of the algorithm is more obvious for classes with a large difference in length and width in the MS COCO minival set. The algorithm proposed in this paper does not change the structure of the detector model, does not increase the number of parameters, brings a significant accuracy improvement with only a small increase in training time, and does not require additional computation during the inference of the model, which does not affect the detection speed. In the next work, further research will be carried out on the regression loss in the CLA, and the aspect ratio of the GT box and the predicted box will be considered in the calculation of the regression loss to further improve the performance.

## Figures and Tables

**Table 1:** Effect of each component in the LADA on the COCO minival set.

EPCP	CLA	DLT	AP	AP50	AP75	APs	APm	APl
			39.96	57.86	43.14	23.47	44.01	52.51
		√	40.26	57.35	43.79	22.21	45.11	53.94
	√	√	40.34	57.56	43.65	22.87	44.98	53.99
√		√	40.36	57.41	44.07	22.59	44.97	53.04
√	√	√	40.46	57.54	43.86	23.12	45.07	54.30

**Table 2:** Effect of center prior type and hyperparameter r on the COCO minival set (FCOS).

Methods		*r*	AP	AP50	AP75	APs	APm	APl
CP	EPCP							
√		1.5	40.19	57.07	43.75	22.55	45.01	53.39
√		2	40.33	57.67	43.87	22.77	44.64	53.36
√		2.5	40.34	57.56	43.65	22.87	44.98	53.99
√		3	40.30	57.43	43.86	23.11	44.84	53.87
	√	1.5	40.21	57.33	43.67	23.24	44.66	53.87
	√	2	40.44	57.62	44.04	23.95	44.86	54.37
	√	2.5	40.46	57.54	43.86	23.12	45.07	54.30
	√	3	40.35	57.39	43.87	23.60	45.14	53.72

**Table 3:** Effect of center prior type on COCO minival set (RetinaNet).

Methods		*r*	AP	AP50	AP75	APs	APm	APl
CP	EPCP							
√		2.5	40.33	57.37	43.89	22.92	44.47	54.41
	√	2.5	40.41	57.68	44.04	23.60	44.99	53.88

**Table 4:** Comparison of various categories of AP values on the COCO minival set.

	FCOS + CP	FCOS + ATSS	FCOS + PAA	FCOS + LADA
bicycle	28.77	29.55	28.86	30.50
motorcycle	39.88	40.86	41.78	42.53
airplane	66.35	63.91	66.17	67.13
train	60.26	62.40	62.38	63.25
fire hydrant	65.03	64.51	65.79	66.84
horse	52.92	54.94	57.60	58.68
cow	55.89	56.35	56.00	57.22
elephant	62.78	64.03	63.91	65.28
giraffe	64.82	67.75	67.01	69.15
snowboard	26.04	25.14	24.22	26.48
tennis racket	44.01	45.21	44.60	47.06
remote	26.73	27.09	26.75	28.30
scissors	28.04	22.88	27.42	28.76

**Table 5:** Effect of EPCP on the COCO minival set (FCOS detector with centerness branch).

Methods	Aux Branch	AP	AP50	AP75	APs	APm	APl
FCOS + CP	centerness	38.70	57.11	41.89	22.34	42.73	49.83
FCOS + EPCP	centerness	38.73	57.28	41.75	22.60	42.87	49.87

**Table 6:** Effect of anchor score programs.

Methods	AP	AP50	AP75	APs	APm	APl
IoU	39.70	57.41	42.97	22.79	43.99	51.02
LA	40.07	57.42	43.40	22.78	44.79	53.92

**Table 7:** Effect of different loss of anchor types.

Anchor Score Program			AP	AP50	AP75	APs	APm	APl
Cls.	Reg.	Dev.						
FL	IoU		40.33	57.23	43.89	23.00	45.03	54.24
FL	GIoU		40.36	57.41	44.07	22.59	44.97	53.04
FL	GIoU	√	40.46	57.54	43.86	23.12	45.07	54.30

**Table 8:** Effect of division methods on the COCO minival set.

Methods	AP	AP50	AP75	APs	APm	APl
PAA* + $\left(N_{pos}=10\right)$	39.96	57.86	43.14	23.47	44.01	52.51
PAA* + $\left(N_{pos}=15\right)$	39.73	58.13	43.02	23.88	44.02	51.85
PAA* + GMM	40.22	58.28	43.38	22.98	44.60	54.04
PAA* + DLT	40.26	57.35	43.79	22.21	45.11	53.94

**Table 9:** Analysis of different values of hyperparameter m on the COCO minival set.

m	AP	AP50	AP75	APs	APm	APl
18	40.23	57.44	43.60	22.57	44.83	53.36
20	40.46	57.54	43.86	23.12	45.07	54.30
22	40.29	57.41	43.77	22.41	44.98	53.68

**Table 10:** Comparison of training costs of different algorithms on the COCO minival set.

Method	AP	GFLOPs	Parameters(M)	FPS	Training Time	$h_{AP}$
RetinaNet	36.35	6.81	7.66	23.82	33.43	1.09
FCOS	38.70	5.17	6.10	25.21	30.88	1.25
ATSS	39.70	5.17	6.10	25.45	30.02	1.32
PAA	40.22	5.17	6.10	25.00	45.93	0.88
LADA	40.46	5.17	6.10	25.45	33.63	1.20

**Table 11:** Performance comparison on the COCO minival set with the FCOS detector.

Method	Aux Branch	AP	AP50	AP75	APs	APm	APl
CP	centerness	38.70	57.11	41.89	22.34	42.73	49.83
ATSS	centerness	39.19	57.03	42.28	22.41	43.26	51.66
ATSS	IoU	39.70	57.41	42.97	22.79	43.99	51.02
PAA	IoU	40.22	58.28	43.39	22.98	44.60	54.04
LADA	IoU	40.46	57.54	43.86	23.12	45.07	54.30

**Table 12:** Performance comparison on the COCO minival set with the RetinaNet detector.

Method	Aux Branch	AP	AP50	AP75	APs	APm	APl
TH(IoU)	centerness	37.12	55.35	39.98	21.16	41.52	48.49
ATSS	centerness	39.19	57.23	42.00	23.59	43.47	51.52
ATSS	IoU	39.67	57.33	42.98	22.79	43.84	52.06
PAA	IoU	40.20	58.28	43.50	22.92	44.53	53.66
LADA	IoU	40.41	57.68	44.04	23.60	44.99	53.88

## Data Availability

Not applicable.
